# Cerebral Palsy and Epilepsy in Children: Clinical Perspectives on a Common Comorbidity

**DOI:** 10.3390/children8010016

**Published:** 2020-12-31

**Authors:** Piero Pavone, Carmela Gulizia, Alice Le Pira, Filippo Greco, Pasquale Parisi, Giuseppe Di Cara, Raffaele Falsaperla, Riccardo Lubrano, Carmelo Minardi, Alberto Spalice, Martino Ruggieri

**Affiliations:** 1 Unit of Clinical Pediatrics, Department of Clinical and Experimental Medicine, AOU "Policlinico", PO "G. Rodolico", University of Catania, 95123 Catania, Italy; ppavone@unict.it (P.P.); dottalep@hotmail.com (A.L.P.); coicoico@hotmail.com (F.G.); 2 Postgraduate Training Program in Pediatrics, Department of Clinical and Experimental Medicine, University of Catania, 95123 Catania, Italy; carmelagulizia@hotmail.it; 3 NESMOS Department of Pediatrics, Sapienza University of Rome, Sant’Andrea University Hospital, 00161 Rome, Italy; pasquale.parisi@uniroma1.it; 4 Department of Pediatrics, University of Perugia, 06132 Perugia, Italy; giuseppe.dicara@unipg.it; 5 Neonatal Intensive Care Unit (NICU), Neonatal COVID-19 Center, AOU “Policlinico”, PO San Marco, University of Catania, 95123 Catania, Italy; raffaelefalsaperla@hotmail.com; 6 Dipartimento Materno Infantile e di Scienze Urologiche, Sapienza Università di Roma, UOC di Pediatria, Neonatologia, Ospedale Santa Maria Goretti, Polo di Latina, 04010 Latina, Italy; riccardo.lubrano@uniroma1.it; 7 Department of Anaesthesia and Intensive Care, University Hospital “G. Rodolico” of Catania, 95123 Catania, Italy; minardi.carmelo@virgilio.it; 8 Child Neurology Division, Department of Pediatrics, Sapienza University of Rome, 00161 Rome, Italy; alberto.spalice@uniroma1.it; 9 Unit of Rare Diseases of the Nervous System in Childhood, Department of Clinical and experimental Medicine, Section of Pediatrics and Child Neuropsychiatry, University of Catania, 95123 Catania, Italy

**Keywords:** cerebral palsy, quadriplegia, hemiplegia, epilepsy, seizures

## Abstract

Cerebral palsy (CP) is a frequent cause of childhood disability often associated with a complex group of disorders, including epilepsy, which is reported to impact approximately 40% of affected individuals. This retrospective study involved a group of children affected by CP, some of whom also had comorbid epilepsy. The aim of this study was to report our experience of analyzing, in particular, (a) some of the clinical aspects of the different type of CP, and (b) the relationship between the clinical data of children affected by CP plus epilepsy and each type of CP. Methods: This retrospective single-center study was performed with 93 children admitted to the Pediatric Department of the University of Catania, Italy, affected by CP and distinguished according to the type of motor clinical presentation, with 46 showing epileptic seizures, compared to a control group of 136 children affected by epilepsy without other neurologic disorders. Results: Among the 93 CP children, 25 (27%) had spastic quadriplegia (plus one patient with dystonic quadriplegia), 39 (42%) had spastic hemiplegia, 11 (12%) had spastic diplegia (plus two with ataxia and one with dyskinetic CP), and 14 (15%) did not have a well-defined type of CP. The frequency of epilepsy was higher in affected CP children who showed major motor dysfunction (GMFCS IV–V types). As regards the 46 children with CP plus epilepsy, compared to the group of the control, the age of epilepsy onset was found to be statistically significant: 21 ± 35.1 months vs. 67 ± 39.7. Conclusions: Epilepsy represents one of the most frequent comorbidities of cerebral palsy. In children with CP, particular attention should be paid to the early identification and treatment of comorbid epilepsy.

## 1. Introduction

Cerebral palsy (CP) is the most common cause of childhood disability and is recognized as a heterogeneous condition. Based on international consensus, a generally agreed upon definition of CP is as follows: CP describes a group of permanent disorders of the development of movement and posture, causing activity limitation, that are attributed to non-progressive disturbances that occurred in the developing fetal or infant brain [1,2]. The motor disorders of CP are often associated with a wide and complex group of disturbances, such as sensation, perception, cognition, communication, and behavior disturbances, as well epilepsy and secondary musculoskeletal problems [3,4,5]. The complex manifestations of the disorder led to an extension of the term CP to cerebral palsy spectrum disorder. The incidence of CP ranges from 1/500 live births [6] to 2–3/1000 live births [7], while the prevalence in Australia has been reported to be 1.4 (95% CI, 1.3–1.5) per live births [2]. Prematurity and low birth weight are the main risk factors for CP. Other risk factors include congenital brain malformations, genetic disorders, hypoxic-ischemic encephalopathy, pre- or perinatal stroke, in vitro fertilization or the use of assisted reproductive technology, kernicterus, maternal disorders of clotting, maternal-fetal infections, multiple gestation, neonatal seizures, neonatal sepsis, pre- or post-neonatal meningitis, traumatic brain injury, and pre-pregnancy obesity [7]. However, it is known that some children who develop CP are born at term without any identified risk factors.

The classification of cerebral palsy is based on various criteria: motor abnormalities and functional motor capacity, anatomical distribution (monoplegia, hemiplegia, diplegia, or quadriplegia) and neuroimaging, associated damage, and the timing of presumed random events (prenatal, perinatal, or postnatal). The clinical phenotypes are characterized by the inability to regulate muscle tone and to perform voluntary movements. The severity of the signs and symptoms may vary from a mild to severe motor impairment. The level of motor dysfunction may be measured by the Gross Motor Function Classification System (GMFCS). Based on clinical findings, CP is generally classified as spastic, dyskinetic, ataxic, or mixed such as ataxic-spastic. Thirty-five percent of children with CP have spastic diplegia, which is the most common clinical phenotype of CP [2,8], which is due to damage to the immature oligodendroglia between 20 and 34 weeks of gestation. In this type of CP, the risk is inversely proportional to gestational age and is mainly associated with premature birth and can be detected with a brain magnetic resonance imaging (MRI), which shows severe periventricular leukomalacia and multicystic cortical encephalomalacia [9]. Some children with spastic diplegia have normal cognitive function and good prognosis for independent ambulation. Spastic hemiplegia has been recorded in 25% of children with CP, and is most commonly seen in infants born at term and, in most cases, is due to in utero or perinatal stroke [3,9,10]. Extrapyramidal CP includes the choreoathetoid, dystonic, or dyskinetic clinical phenotypes and comprises 15% CP cases. In this group, infants born at term are the most affected, and hypoxic-ischemic encephalopathy, kernicterus, neurometabolic, or neurogenetic disorders are the most reported etiologic events [11]. The diagnosis of CP is primarily based on clinical findings and is generally more reliable after two years of age, because early signs and symptoms suggestive of CP may, in fact, be a normal variation or developmental lag, which tend to resolve in many infants [8]. The persistence of primitive reflexes or primary motor patterns beyond the expected age is a key clinical characteristic of CP and a diagnosis of CP is first suspected when there is a failure to attain certain key milestones at the expected age. The presence of brain abnormalities, mostly based on structural MRI, is well established in the vast majority of children with CP [12]. White matter lesions have been identified as the most common type of brain lesion, primarily causing spastic CP, including diplegia (46.4%), hemiplegia (33.2%), and spastic diplegia (13.2%) [13]. Epilepsy is a clinical entity often associated with CP. According to the ILAE classification of epilepsy, the seizure type can be distinguished as focal, generalized, and combined generalized and focal, or as unknown. The diagnosis of epilepsy is based on family and personal history, semiology, and the characteristics of clinical events, with electroencephalogram being the diagnostic gold standard [14].

The aim of this single-center retrospective study was to report some of clinical data in children affected by CP, including the different types. Additionally, statistical differences between CP epileptic children vs. epileptic children without other neurologic disorders, considering sex, age of onset of epilepsy, main types of epilepsy, response to treatment, and outcomes, are reported.

## 2. Methods

The clinical data of 93 children with cerebral palsy, among which 46 had comorbid epilepsy, observed at the Pediatrics Department of the University of Catania, Italy, from May 2008 to November 2018, were retrospectively collected. The types of CP were distinguished, and gestational age, birth weight, frequency and age onset of epilepsy, and the results of the neuroimaging type were reported according to the motor dysfunction. Moreover, among the group of 46 with CP plus epilepsy, the clinical data were compared to a control group of 136 children affected by epilepsy observed in the same period without any other neurologic impairment. The group of poorly defined CP included those patients in whom it was not possible to attribute a precise type of motor alteration. The differences among the two groups concerning the age of onset of epilepsy, the types of epilepsy, the response to treatment, and the outcome of epileptic seizures were analyzed. The statistical analysis was conducted using the SPSS (version 18), with the chi-square calculation for categorical variables and the Mann–Whitney test for continuous variables.

## 3. Results

### 3.1. Types of Cerebral Palsy and Related Clinical Data

#### 3.1.1. Types of Motor Dysfunction

**Figure 1:**
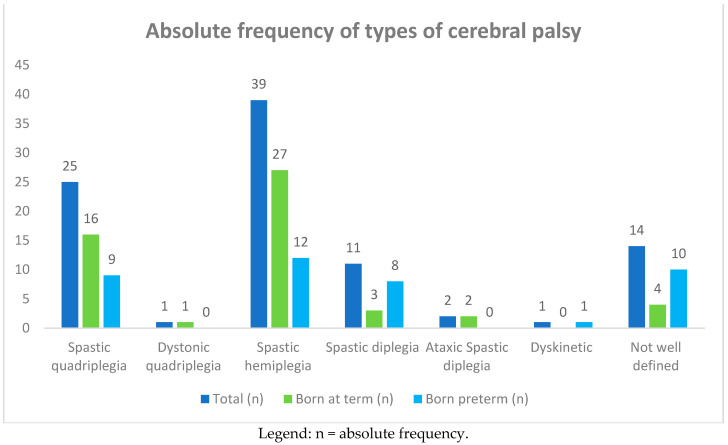
Absolute frequency of types of cerebral palsy (CP) in 93 affected children born at term and preterm.

**Figure 2:**
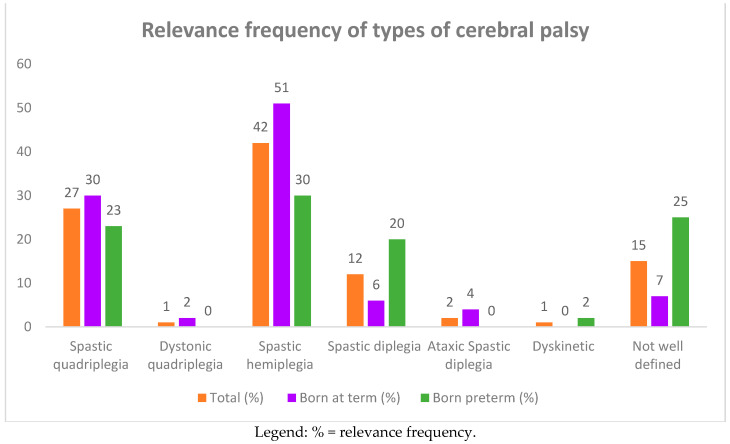
Relevance frequency of types of cerebral palsy (CP) in 93 affected children born at term and preterm.

Ninety-three children with cerebral palsy 44 (47%) males and 49 (53%) females were recruited. The ages of the children at the time of this study ranged from 1 to 10 years, with an average of 10 ± 5 years. Among the 93 children, 25 (27%) had spastic quadriplegia and one had dystonic quadriplegia, 39 (42%) had spastic hemiplegia, 11 (12%) had spastic diplegia, two (2%) had ataxic spastic diplegia, one had dyskinetic CP, and 14 (15%) had a poorly defined type of CP (see Table 1, and Figure 1 and Figure 2). The subjects suffering from quadriplegia and hemiplegia were mostly born at term (30% and 51% vs. 23% and 30% of preterm births, respectively), while poorly defined, dyskinetic, and spastic diplegia CP were more frequent in the group of preterm births (25%, 2%, and 20%).

#### 3.1.2. Gestational Age and Birth Weight

Gestational age and birth weight averaged 36.4 ± 4 weeks and 2665 ± 792.8 g, respectively. A statistically significant difference was observed in the hemiplegic group compared to the diplegic children regarding gestational age (38 ± 2.8 vs. 34.4 ± 4.7; *p* = 0.014) and birth weight (2919.9 ± 629.8 g vs. 2421.8 ± 731.9 g; *p* = 0.028).

#### 3.1.3. Type of CP and Frequency of Epilepsy

**Figure 3:**
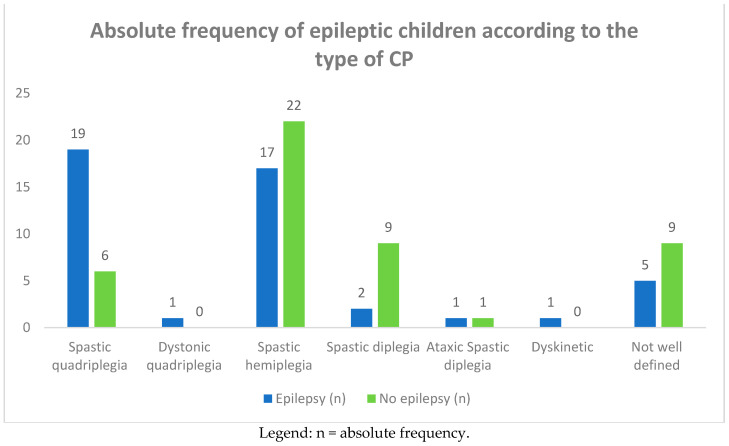
Absolute frequency of epileptic children according to the type of CP.

**Figure 4:**
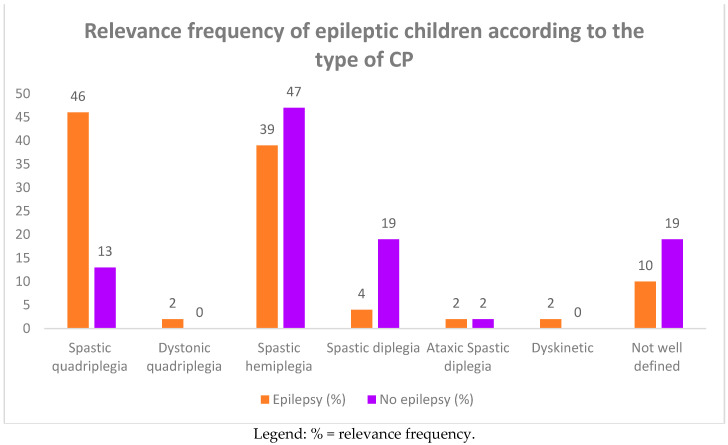
Relevance frequency of epileptic children according to the type of CP.

Among the group of 93 CP children, 46 of them (49.4%) complained of epileptic seizures. In Table 2 and Figure 3 and Figure 4, we report the frequency of epilepsy according to the type of CP. The frequency was higher in term infants than in preterm infants (61% vs. 39%), affecting females more (26 females vs. 20 males). Epilepsy was also more frequent in subjects suffering from spastic quadriplegia (19 cases, 46%) compared to the other forms of CP (spastic hemiplegia: 17 cases, 39%; spastic diplegia: two cases, 4%).

Furthermore, in the comparison between children with spastic quadriplegia and those with spastic hemiplegia, the frequency of epilepsy was significantly higher in the former (46% vs. 39%; *p* = 0.02). As in the comparison with the children with spastic diplegia, the frequency was significantly higher in the quadriplegic children (46% vs. 4%; *p* = 0.002). Instead, no statistically significant difference was found in the comparison between hemiplegic and diplegic children.

#### 3.1.4. The GMFCS levels were performed in 28 CP children and, among these, in 12 CP epileptic children (Table 3). Comparison between the different levels of GMFCS and frequency of epilepsy was higher in children with IV–V levels of motor dysfunction.

#### 3.1.5. Age of Onset of Epilepsy

Thirty-two patients (69.5%) had the first seizure within the age of one year. On average, the age of onset of seizures was 21 ± 35.1 months, within the first two years of life.

#### 3.1.6. Types of Epilepsy

The types of epilepsy diagnosed in the group of children with cerebral palsy were focal then generalized seizures, focal seizures, West syndrome, neonatal seizures, and epileptic encephalopathy (Table 4). In spastic quadriplegia, the most commonly observed types of epilepsy were focal then generalized epilepsy and epileptic encephalopathies, in which the highest incidence of drug resistance was reported. In the cases of hemiplegia, focal epilepsy that typically involved the side affected by the paresis was more frequent. In children with spastic quadriplegia, one child started with West syndrome, three children presented neonatal seizures (in one case, proceeding onto West syndrome), and subsequently focal-generalized tonic-clonic epilepsy. In the group of children with spastic hemiplegia, two started as neonatal seizures and then manifested West syndrome and two who started with West syndrome subsequently developed focal and then generalized tonic-clonic epilepsy. Finally, in the group of children poorly defined CP, one case started with neonatal seizures and then presented focal-generalized epilepsy.

#### 3.1.7. Neuroimaging

The most common brain lesions reported in the 38 CP children were encephalomalacia with pore encephalic cysts, alterations of the white matter from hypoxic-ischemic outcomes, brain malformations (alterations of the corpus callosum, polymicrogyria, and microcephaly), or perinatal thrombotic events. In subjects with quadriplegia, the highest percentage of brain malformations was found compared to the other groups of CP: an association with microcephaly, thinning of the corpus callosum, and alterations of the white matter were found in 4/38 children; hypoplasia of the corpus callosum was reported in 4/38 children associated with other anomalies (triventricular hydrocephalus, encephalomalacia, and pachygyria); encephalomalacia was found in 9/38 patients, one of which was associated polymicrogyria. In the diplegic group, signs of white matter anomalies were found in 5/9 children, holoprosencephaly in one child, and thinning of the corpus callosum with signs of periventricular leukomalacia in two children. Outcomes of ischemic infarct were prevalent in cases of hemiplegia as a consequence of perinatal thrombosis (28/38 patients). In the remaining cases, signs of white matter damage from hypoxic-ischemic outcomes and neuronal migration defects (pachygyria and micropolygyria) were described, and in one case, there was severe cerebral atrophy with microcephaly and the thinning of the corpus callosum.

#### 3.1.8. Neuroimaging: Brain Malformations and Perinatal Damage

Lesions identified by magnetic resonance imaging were compared to look for a possible correlation between the presence of epilepsy and brain malformations or perinatal lesions. No significant differences were found either between CP epileptic and non-epileptic children or between the group of CP children with epilepsy and congenital brain malformations and the group of CP children with epilepsy and perinatal damage.

### 3.2. Clinical Data of CP Epileptic Children Compared to Epileptic Children without Other Cerebral Anomalies

#### 3.2.1. Sex and Age of Onset of Seizures

In the 46 epileptic children with CP compared to the group of controls, no differences in gender (44% male and 56% female vs. 54% male and 46% female) were found. Regarding the age of onset of epilepsy, a statistically significant difference was found between the two groups: 67 ± 39.7 months in the control group and 21 ± 35.1 months in the CP epileptic children (*p* = 0.0001).

#### 3.2.2. Main Types of Epilepsy

The main types of epilepsy found in the control group were focal seizures (48, 35%), focal then generalized tonic-clonic seizures (36, 26%), and the absence of epilepsy (24, 18%). In lower percentages, myoclonic epilepsy (3%), epilepsy with centrotemporal spikes epilepsy (3%), and neonatal convulsions, epileptic encephalopathy, atonic seizures, and photosensitive epilepsy (all affecting 1.5%) were also found.

#### 3.2.3. Antiepileptic Treatment: Monotherapy vs. Polytherapy

The use of antiepileptic drugs in mono- or polytherapy was compared between the two groups. In addition to first-line drugs such as valproic acid, topiramate, phenobarbital, levetiracetam, second-line drugs were associated (i.e., vigabatrin, lamotrigine, clonazepam, clobazan, and gabapentin). Out of the 46 epileptic children with CP, 21 were receiving politherapy, 16 monotherapy, seven were not receiving any therapy, and two children were lost to follow-up. Analyzing, in detail, the individual groups, of the 17 quadriplegic subjects treated, 12 (70%) were in polytherapy and six (30%) in monotherapy, and 7 diplegic patients were in monotherapy; of the 13 hemiplegic children, nine (69%) were in monotherapy and four (31%) in polytherapy. This shows that the use of associated antiepileptic drugs is more frequent in the group of quadriplegic children. In the control group, of the 121/136 treated, 100 patients (83%) were on monotherapy and 21 (17%) were in polytherapy. In the comparison between epileptics with CP and the control group, a significant difference was found regarding the use of drugs in polytherapy, most frequently used in the former group (*p* = 0.005).

#### 3.2.4. Outcome of CP Epileptic Children

Of the 46 epileptic children with CP, we observed that approximately half (54%) achieved quite satisfactory seizure control. Despite the polytherapy, three children continued to experience epileptic seizures and, therefore, the dosage of the drugs had to be increased. These children were all affected by spastic quadriplegia; two were born full term and of adequate weight, while there was an extremely premature infant. In four children, antiepileptic treatment was gradually suspended due to the resolution of symptoms.

## 4. Discussion

Cerebral palsy is not a disease entity in the traditional sense, but rather a clinical description of children who share features of non-progressive brain injuries or lesions acquired during the ante-nnal, perinatal, or early postnatal period [1]. It can manifest itself in several ways, mainly as spastic, athetoid, or ataxic palsies; moreover, it is one of the most common causes of motor disability in children and is frequently associated with other problems, such as developmental delays, sensory defects, and epilepsy. The clinical management of children with CP is directed toward maximizing function and participation in activities and minimizing the effects of the factors that can make the condition worse, such as epilepsy, feeding challenges, hip dislocation, and scoliosis [15]. The significance of epilepsy in individuals with CP is discussed controversially in the literature. Epilepsy is common in cerebral palsy, present in 30%–40% of cases [4,16], and seizures are reported on 35.5% of CP children in the hemiplegia subgroup [15]. In the 93 children analyzed in the present study, the incidence of epilepsy was calculated to be 49%, on average higher than that reported in the literature [16,17,18]. The difference in the frequency of CP-epilepsy association may depend on the selection of the cases. Sellier et al. [17] maintained that epilepsy is more frequent in children presenting with dyskinetic- or bilateral spastic-type CP and when other associated impairments are present. Moreover, the frequency of epilepsy was greater in the group of subjects suffering from spastic quadriplegia, and with higher motor dysfunction, as reported in the literature [16]. In the present cases, the types of epilepsy most frequently observed in CP children were focal-generalized (37%), epileptic encephalopathy (15%), and seizures with neonatal onset (33%), which were mainly found in children with spastic quadriplegia, focal in the study of Tillberg et al. [4]. Epilepsy in CP is related to underlying brain lesions and can be classified according to the anatomical site of the brain lesion; namely, the cerebral cortex, pyramidal tract, extrapyramidal systems, or cerebellum [11]. The neurodevelopmental outcomes are, however, predominantly due to underlying brain lesions. In terms of children with CP, grey matter lesions are more common. As suggested by El-Tallawy et al. [18], neonatal seizures can be a strong predictor of epilepsy in cerebral palsy. Sadowska et al. [19] investigated 181 children with CP and selected 102 children affected by epilepsy (56.35%); among this group, 44 (43%) had drug-resistant epilepsy and only 15 (14.71%) were responsive to treatment. Epilepsy was more frequent in CP children with quadriplegia (75%), ataxia (83%), and mixed forms (80%) in comparison to diplegia (32%) and hemiplegia (38%). Maternal hypertension is considered to be a relevant risk factor for epilepsy in CP children, for which Sadowska et al. [19] reported in their study an odds ratio (OR) of 12.46 (*p* = 0.0001), while for drug-resistant epilepsy, they reported an OR of 9.86 (*p* = 0.040) [19]. Other risk factors are represented by delivery by cesarean section, for which the risk of epilepsy in CP children is double (OR = 2.17, *p* = 0.0129), neonatal seizures with a risk for CP epilepsy calculated as an OR of 3.04 (*p* = 0.011), and drug-resistant epilepsy with an OR of 4.02 (*p* = 0.002). In this study, a higher incidence of epilepsy in CP children borns at term compared to preterm children and in females rather than males was reported. According to our results, quadriplegia and hemiplegia were mostly found in children born at term, while diplegia was found to be more prevalent in preterm children. Epilepsy had a greater impact in those borns at term than in preterms (61% vs. 39%) children. The clinical data of the 46 epileptic CP children compared to the control group of 136 epileptic children without any other associated neurological disorders showed that the onset of seizures was earlier in the epileptic children with CP than in the control group (*p* = 0.0001). Meanwhile, the onset of epileptic seizures was found to be, on average, within the second year of life in children with CP, while in the control group, the onset was around the age of five years. All children who developed neonatal seizures, or epilepsy later on, showed both white and grey matter injuries, as reported in the study of Cooper et al. [13]. With regard to antiepileptic treatment, which has been performed by a group of the same institution a significant difference was found between the two groups, as polytherapy treatment was the most used treatment in epileptic CP children (25% vs. 3%, respectively; *p* < 0.05). In the present analysis, the size of the cerebral injury also had a relevant role in the response to drug epileptic treatment. Seizures of the children with the most extensive injuries occurred in drug-resistant therapy, as also reported in the study of Reid et al. [12]. Polytherapy was mainly performed in CP epileptic children who had important motor involvement (quadriplegic children) and higher motor dysfunctions and as the outcome of cerebral malformation. Most of the children of this study had an accurate neurological follow-up and quite satisfactory seizure control. Particular attention has been given to growth and bone metabolism in the treatment of the epileptic children as it is well known that antiepileptic drugs and polytherapy in particular can cause a significant increase in bone turnover, decreased bone density and increased risk of fractures in treated children [20,21]. 

## 5. Conclusions

Children with cerebral palsy, epilepsy, and cognitive disability constitute a wide concern in pediatric neurology. The management of children with CP is complicated and requires multidisciplinary management in order to improve the quality of life of these children. Their associated comorbidities are many, one of the most important being epilepsy, which causes disabilities and difficulties in management. Antiepileptic polytherapy is the gold standard of clinical management in epileptic CP children and preferred even if complete seizure control is not always achieved. Yet, with the increased risk of antiepileptic drug-associated side effects, it should be used with caution in these children to balance the risks and benefits of the administered therapies. Only a close and constant multidisciplinary collaboration allows for good management of children affected by this common disorder. 

## Figures and Tables

**Table 1:** Types of cerebral palsy (CP) in 93 affected children born at term and preterm.

Type of Cerebral Palsy	Total		Born at Term		Born Preterm	
	Absolute Frequency	Relative frequency %	Absolute Frequency	Relative Frequency %	Absolute Frequency	Relative Frequency %
Spastic quadriplegia	25	27	16	30	9	23
Dystonic quadriplegia	1	1	1	2	-	-
Spastic hemiplegia	39	42	27	51	12	30
Spastic diplegia	11	12	3	6	8	20
Ataxic spastic diplegia	2	2	2	4	-	-
Dyskinetic	1	1	-	-	1	2
Poorly defined	14	15	4	7	10	25
Total	93	100	53	57	40	43

**Table 2:** Frequency of epilepsy in the children according to the type of CP.

Type of Cerebral Palsy	Epilepsy		No Epilepsy	
	Absolute Frequency	Relative Frequency %	Absolute Frequency	Relative Frequency %
Spastic quadriplegia	19	46	6	13
Dystonic quadriplegia	1	2	-	-
Spastic hemiplegia	17	39	22	47
Spastic diplegia	2	4	9	19
Ataxic spastic diplegia	1	2	1	2
Dyskinetic	1	2	-	-
Poorly defined	5	10	9	19
Total	46	49	47	51

**Table 3:** GMFCS Levels and frequency of epilepsy carried out in 28 affected children.

		Epilepsy		No Epilepsy	
GMFCS Levels	Children	Absolute Freq. Relative Freq %		Absolute Freq. Relative Freq %	
**GMFCS I**	2	-	-	2	7.14%
**GMFCS II**	4	1	3.57%	3	10.71%
**GMFCS III**	2	1	3.57%	1	3.57%
**GMFCS IV**	13	5	17.86%	8	28.57%
**GMFCS V**	7	5	17.86%	2	7.14%
**Total**	28	12	42.86 %	16	57.13%

**Table 4:** Types of epileptic seizures selected according the type of CP.

Types of Seizures	Spastic Quadriplegia	Distonic Quadriplegia	Hemiplegia	Spastic Diplegia	Spastic Ataxic Diplegia	Dyskinetic Cerebral Palsy	Poorly Defined Cerebral Palsy
Focal then Generalized	9	0	5	1	0	0	2
Focal	0	0	6	1	1	0	0
West syndrome	2	0	4	0	0	0	0
Neonatal seizures	5	1	4	0	0	1	4
Epileptic encephalopathy	7	0	0	0	0	0	0

## Data Availability

The data used to support the findings of this study may be released upon application to the corresponding author, who can be contacted at ppavone@unict.it.
